# A rare case report of Herlyn-Werner-Wunderlich syndrome: Unraveling unusual urinary anomalies and literature review

**DOI:** 10.1016/j.heliyon.2024.e33558

**Published:** 2024-06-25

**Authors:** Xiaotong Xu, Yanpeng Tian, Jingwen Zhou, Zhongkang Li, Li Meng, Xianghua Huang, Mingle Zhang

**Affiliations:** a Department of Gynecology, The Second Hospital of Hebei Medical University, Shijiazhuang, Hebei, 050000, PR China; b Department of Gynecology, The First Affiliated Hospital of Zhengzhou University, Zhengzhou, Henan, 450052, PR China; c Department of Gynecology, Loujiang New City Hospital of Taicang, Suzhou, Jiangsu, 215334, PR China

**Keywords:** HWWS, Gartner's duct cyst, Classification, Treatment, Case report

## Abstract

Herlyn-Werner-Wunderlich syndrome (HWWS) is a rare congenital genitourinary abnormality defined by uterine didelphys, obstructed hemivagina, and ipsilateral urological anomalies. Accurate diagnosis and prompt commencement of therapy can be difficult owing to heterogeneous genitourinary malformation among different patients. This is a case report of a patient with rare HWWS with uterine didelphys, obstructed hemivagina, vagina-ureteral remnant fistula (Gartner's duct cyst), and ipsilateral kidney dysgenesis who complained of intermittent abdominal pain during menstruation. The right ureteral remnant of the patient was distinctive, with three portions. The upper section was connected to the right dysplastic kidney, the lower section formed the fistulous tract with the vagina and bladder, while the middle section communicated with Gartner's Duct Cyst, which merged with the vagina and opened to the posterior cavity of hemivagina. The lower section of the right ureter was excised and ligated during laparoscopic surgery, while the upper section was excised. The patient recovered after surgery. We presented this rare case and conducted a literature review to provide a more comprehensive understanding of HWWS. This could help gynecologists effectively reduce misdiagnosis and missed diagnosis, especially when combined with complicated urinary malformation.

## 1 Introduction

The Herlyn–Werner–Wunderlich syndrome (HWWS), also known as obstructed hemivagina and ipsilateral renal agenesis syndrome and oblique vaginal septum syndrome, is characterized by uterine didelphys, obstructed hemivagina, and urological malformations [1]. HWWS has an incidence rate of approximately 0.1%–3.8 % [2]. Research has reported that 74%–95.4 % of patients with HWWS have kidney dysplasia [3], with 55%–70 % presenting with unilateral kidney agenesis [4]. Besides, uterine septum, bicornuate/unicornuate uterus, ipsilateral ectopic ureter, multicystic dysplastic kidney (MCDK), and Gartner's duct cyst have also been reported [2,5]. However, the etiology and pathogenesis of HWWS with urological anomalies remain poorly understood.

In this study, we reported a case of HWWS with a rare urinary malformation presenting as uterine didelphys, obstructed hemivagina, Gartner's Duct Cyst, vagina-ureteral remnant-bladder fistula, and ipsilateral kidney dysgenesis. According to the rare case and literature review, we hope to expand our knowledge of the complexity of HWWS with rare urinary malformations.

### 1.1 Case presentation

**Fig. 1:**
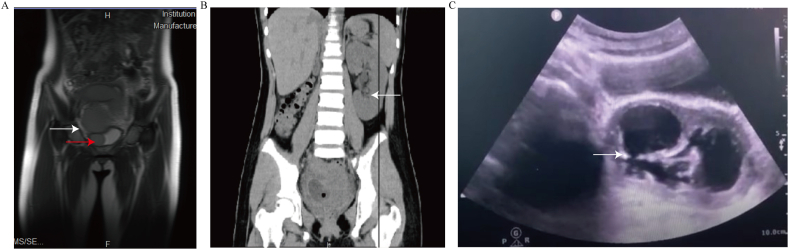
MRI, CT, and transabdominal ultrasound manifestations of the patient. (A) The right ureteral remnant partly forms a fistula tube communicating with the vagina and bladder (white arrow). The enlarged part of the ureteral remnant merged with the vagina (red arrow). (B) The right kidney agenesis and left kidney enlarged (white arrow). (C) Two vaginal oblique septa. The inner part communicates with the outer one through a valve-like structure (white arrow).

A 10-year-old girl who experienced menarche three months ago reported experiencing intermittent abdominal pain during menstruation for two months and aggravated for seven days, combined with dysuria, was admitted to our hospital. Physical and anorectal examinations of the patient revealed no apparent anomalies. Ultrasound examination indicated uterine didelphys, a double cervix, and a pelvic cyst. Then, pelvic magnetic resonance imaging (MRI) was performed to investigate complicated malformations. The MRI exhibited uterine didelphys, double cervix, right hematometra, hematocervix, hematosalpinx, hematometrocolpos, and a strange pelvic cyst communicating with a strip-type tube (between the bladder and the right vaginal wall) (Fig. 1A). The right ovary and left adnexa revealed no apparent anomalies. Abdominal and pelvic computed tomography (CT) detected the absence of the ipsilateral kidney and enlarged contralateral kidney (Fig. 1B). The patient was diagnosed with a rare variant of HWWS based on the imaging outcomes. However, the precise anatomy of this rare variant remains unclear. Accordingly, a two-stage surgical procedure was performed.

During stage-I surgery, the patient underwent hysteroscopy and cystoscopy to incise into hemivagina and place urinary Foley catheters in the vaginal septum cavity and bladder to relieve the obstruction. The hysteroscopy and cystoscopy findings confirmed the malformation structure. The surgical procedure was as follows. Initially, we performed a cystoscopy in the lithotomy position. The bladder contains old blood clots and blood flows into the bladder from the pelvic bloody cyst. This revealed that the strip-type tube between the bladder and vagina may be a fistula that opens into the bladder. However, it is unclear whether the fistula is the right ureter. During hysteroscopy, a complete vagina septum and the left cervix were observed. We made an incision in the vaginal septum under ultrasound monitoring and found two vaginal oblique septa. Two anechoic cystic cavities were identified in the superior vaginal segment. The inner part communicated with the outer one via a valve-like structure (Fig. 1C). Uterine didelphys, the right uterine cavity, and fallopian tube hematocele were also identified on ultrasound examination as preoperative imaging data. Entering the hole of the inner incomplete septum, the right cervix was observed, but the other fistula opening failed to be found due to numerous old blood clots. Then, one Foley catheter was introduced into the inner septum cavity, while the other was placed into the bladder to drain the right hematometra, hematocervix, hematosalpinx, hematocolpos, and pelvic cyst.

**Fig. 2:**
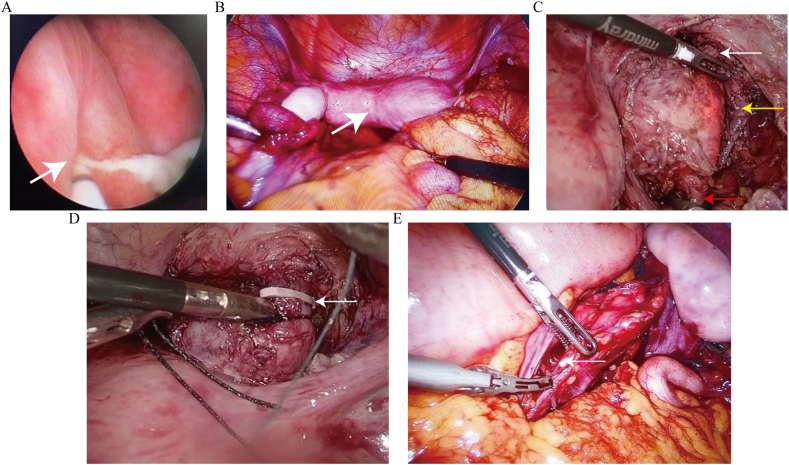
Cystoscopy and laparoscopy (planform) manifestations in the patient. (A) The right ureter orifices are covered by purulent secretion (white arrow). (B) Uterine didelphys (white arrow). (C) Light illuminated using urethroscopy. The malformation of the right ureter remnant is divided into three sections: the lower section formed a fistula tube connecting the vagina and bladder (white arrow), the middle section merged with the vagina (yellow arrow) and presented as a cystic mass on MRI (Fig. 1), and the upper section leading to the dysgenesis kidney (red arrow). (D) The descending section of the right kidney remnant was excised and ligated (white arrow). (E) The blind end of the right kidney remnant was proved as the right dysgenesis renal by postoperative pathology (white arrow).

**Fig. 3:**
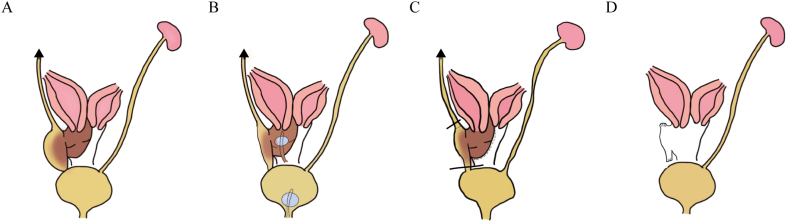
Schematic anatomy and surgical procedures for this variant. (A) Schematic representation of the anatomy. (B) A Urinary Foley catheter was placed to relieve obstruction without impairing the natural structure. (C) The merged section was preserved, the lower section was cut off, and the upper section was excised. The subsequent vaginal septum excision was also performed.

Stage-II surgery was performed 23 days after stage-I surgery because the patient's parents refused stage-II surgery immediately, despite thorough counseling with their parents. During this period, the patient had menstruation combined with fever and mild abdominal pain. Additionally, she complained of blood outflow through two Foley catheters. Three days after menstruation, the patient underwent stage-II surgery. The surgical procedures are as follows. Initially, cystoscopy was performed, identifying two ureter orifices in the bladder, with the right one (the opening of the fistula observed in stage-I surgery) covered by purulent secretion (Fig. 2A). Then, urethroscopy entered into the right ureter, revealing the presence of a blind-ending ureter. Additionally, a fistula was observed on the right wall of the right ureter, approximately 3 cm from the bladder. The inner oblique vaginal septum cavity was connected to the right ureter through the fistula. Afterward, the ureteral stent was placed here as an indicator. Subsequently, laparoscopy displayed uterine didelphys and right pyosalpinx. The bilateral ovary and left fallopian tube appeared normal (Fig. 2B). We opened the right side peritoneal among the bladder, uterus, and vagina to directly visualize the right urogenital tract. The genitourinary malformations were identified using urethroscopy (Fig. 2C). The right vaginal vault protruded towards the right pelvic. The right ureter was divided into three sections: the middle section was approximately 5 cm long, merged with the vagina, and opened to the posterior cavity of the vaginal oblique septum; the lower section formed a fistula approximately 3 cm long, connecting the vagina and bladder, and the upper section was approximately 15 cm long with a blind-ending. Although treatment for Gartner's duct cyst is generally complete excision, it is unsuitable for this case. Given that total resection of the Gartner's duct cyst is required to separate the merged section of the vagina, vagina the wound may be too large to heal, leading to menstruation flowing into the abdominopelvic cavity and possibly influencing sexual intercourse and pregnancy in the future. Therefore, we reserved the merged section, cut it off, ligature the lower section (Fig. 2D), and excised the upper section (Fig. 2E). A surgery team successfully performed cyst marsupialization in two cases of Gartner's duct cyst, supporting our approach. Considering that the patient was an adolescent, a subsequent vaginal septum excision was performed under hysteroscopy. The reserved middle section of the right ureter and the vagina were repaired. The excised ureter was proven to contain tubular components. The final diagnosis was confirmed as HWWS with uterine didelphys, obstructed hemivagina, Gartner's duct cyst, a vagina-ureteral remnant-bladder fistula, and ipsilateral kidney dysgenesis. Fig. 3 presents the schematic anatomy of this case (Fig. 3A–D). Following surgery, the patient recovered and was discharged after three days of treatment with macrolide antibiotics to control the infection. No complications were recorded during the one-year follow-up.

## 2 Discussion

HWWS was first described in 1922 and comprehensively detailed by Herlyn and Werner in 1971 [6,7]. HWWS is defined as uterine didelphys, obstructed hemivagina, and urological anomalies. The etiology and pathogenesis of HWWS with urological anomalies remain poorly understood. We reported a rare variant of HWWS and reviewed the literature to expand our knowledge of the complexity of HWWS with rare urinary malformation.

### 2.1 Embryology

Development of the female reproductive system is a complicated and delicate process. Previous studies indicated that the kidney and ureter are derived from the mesonephric duct. During the first four weeks of embryonic development, defective unilateral mesonephric ducts and attached ureteric buds may cause ipsilateral kidney and ureter agenesis or dysplasia [8,9]. The distal portions of the mesonephric ducts and attached ureteric buds typically move downward to join the primitive bladder posterior wall, eventually forming the definitive ureters, trigone, and bladder neck [10]. Additionally, the mesonephric duct plays a guiding effect in developing the paramesonephric duct [8,9]. If the unilateral mesonephric duct develops agenesis or dysplasia, it can lead to urinary malformations such as unilateral kidney agenesis/dysplasia, MCDK, ectopic ureter, and Gartner's Duct Cyst. Therefore, an abnormal unilateral mesonephric duct may prevent the ipsilateral Müllerian duct from fusing with the contralateral Müllerian duct, leading to distinct development of the bilateral Müllerian ducts. The affected Müllerian duct deviates from the midline at nine weeks of embryonic development, preventing it from connecting and fusing with the center of the genitourinary sinus, and forms a blind duct [8,9], resulting in uterine didelphys, vaginal septum, and uncommon bicornuate/unicornuate uterus. During embryological development, the mesonephric ducts degenerate in females, while the Müllerian ducts differentiate into the fallopian tubes, uterus, and vagina [11,12]. Urogenital malformations result from disruptions in mesonephric duct degeneration and/or Müllerian duct fusion.

### 2.2 Symptoms of HWWS

Since malformations of HWWS can be extremely complicated, HWWS symptoms are diverse. The onset symptom of HWWS is closely associated with its anatomical structure. Patients with a completely obstructed hemivagina or cervicovaginal atresia usually complain of dysmenorrhea, abdominal pain, pelvic mass, fever, or dysuria soon after menarche. It has been reported that the main HWWS symptoms included dysmenorrhea (53.8 %), abnormal uterine bleeding (28.9 %), vaginal discharge (26.6 %), and abdominal pain (15.5 %) [2]. Additionally, endometriosis rates have been indicated to be 13.6%–35 % among women with ureteral obstruction [3,[13], [14], [15], [16], [17], [18], [19]]. However, a recent study analyzed 1673 cases of HWWS and indicated an endometriosis rate of 9.6 %, consistent with the general population [2]. Common urinary symptoms comprised urinary tract infections, hesitancy, incontinence, and dysuria. Meanwhile, the digestive symptoms comprise fecal impaction and diarrhea. In rare cases, conditions like kidney dysplasia with ectopic ureter or Gartner's duct cyst can lead to symptoms, including continuous vaginal drainage following vaginal septum resection [20]. Intermittent mucopurulent discharge, irregular vaginal bleeding, and pelvic inflammatory disease are more prevalent in people with incompletely obstructed hemivagina or duplicated cervix, with limited communication between them. Symptoms typically manifest late, possibly years after menarche [18]. The ipsilateral dysplastic kidney may produce urine in patients combined with the ureter communicating with the cervix/vagina. When the reproductive system simultaneously communicates outward, their initial symptoms might be an abnormal vaginal discharge of clear fluid before menarche, which is called ureteral incontinence [21,22]. In this case, the ectopic ureter communicated with Gartner's duct cyst and a completely obstructed hemivagina, causing dysmenorrhea as the initial symptom. Additionally, the lower section of the ureter formed a fistula between the vagina and bladder, allowing blood to flow into the bladder from Gartner's duct cyst. However, why was hematuria not present at menarche, and why did blood flow into the bladder during cystoscopy? This can be explained by the similar HWWS case reported previously [5]. The Gartner's duct cyst might communicate with the vagina, but not with the bladder, at birth. With the hemorrhage accumulation, increased pressure makes the cyst break and blood flow into the bladder. In summary, the symptoms of HWWS are diverse and closely associated with anatomical structure. The results are presented in Table 1.

**Table 1:** The characteristics of HWWS.

		(%)
Symptoms	n = 1559	
Dysmenorrhea	838	53.8
Urinary problems	43	2.8
Abdominal pain	242	15.5
Abnormal uterine bleeding	451	28.9
Vaginal discharge	353	26.6
Infertility	79	5.1
Dyspareunia	6	0.4
Digestive problems	22	1.4
Pelvic mass	142	9.1
Asymptomatic	179	11.5
Unreported	114	
Classification	n = 1248	
1.1	583	46.7
1.2	28	2.2
2.1	489	39.2
2.2	148	11.9
Unreported	425	
Urologic anomalies	n = 1644	
Ipsilateral renal agenesis	1530	93.1
Ipsilateral renal dysplastic/multicystic	77	4.7
Other renal anomalies	17	1.0
Normal	21	1.3
Ureteral anomalies	92	5.6
Unreported	29	
Endometriosis	161	9.6

### 2.3 The diagnosis of HWWS

Diagnosing congenital defects remains an ongoing challenge in clinical practice due to limitations in earlier categorization systems and inconsistent usage of diagnostic procedures with differing levels of accuracy. Various noninvasive diagnostic methods are available to detect anatomical anomalies in the female genital tract. In 2016, the European Society of Human Reproduction and Embryology (ESHRE)/European Society for Gynecological Endoscopy (ESGE) system suggested a shared agreement on diagnosing female genital abnormalities [23]. Pelvic ultrasound examination and MRI are important diagnostic approaches. CT scanning is no longer recommended due to poor depiction [23]. A gynecological examination and two-dimensional ultrasound (2D-US) are recommended to assess asymptomatic females. Three-dimensional ultrasound (3D-US) can diagnose female genital malformations in “symptomatic” patients at high risk for such anomalies and in asymptomatic females suspected of having an anomaly during standard examination. The ultrasonographic characteristics of these conditions include uterine anomalies (didelphic/bicornuate uterus) with or without uterine effusion, an area without echoes beneath one cervix (sometimes with dense dot-like hyperechoic regions in the echo-free area), and ipsilateral kidney agenesis with compensatory hypertrophy of the contralateral kidney. The paravaginal mass centesis revealed the presence of accumulated pus or blood. MRI and endoscopic assessments like hysteroscopy (HSC) and laparoscopy (LSC) are recommended for patients with suspected intricate abnormalities or diagnostic uncertainties. MRI, coupled with the multiplanar acquisition of images, offers enhanced insights into uterine morphology, continuity with each vaginal lumen, and the characteristics of fluids within these cavities. Imaging techniques can also detect related pathologies like endometriosis, pelvic inflammation, adhesions, and kidney abnormalities [24]. Although CT was used to screen the urinary system in the described case, it is not advised for diagnosing syndromes due to its low accuracy and radiation exposure [23,25]. When MRI is inaccessible or the pictures obtained are unclear, HWWS can be verified and treated using colpo-hysteroscopy. Furthermore, the LSC can be used to identify complex abnormalities. The HSC provides a highly objective assessment of the vagina, cervix, and uterus. HSC and LSC are extremely reliable methods to examine the female genital tract anatomy and are the most accurate diagnostic tools available. Adolescents with symptoms of a female genital abnormality require comprehensive evaluation, including 2D US, 3D US, MRI, and endoscopy. X-ray hysterosalpingography and hysterosalpingo-contrast sonography can provide supplementary details regarding uterine cavity anatomy [23].

In this complicated case, pelvic MRI displayed uterine didelphys, double cervix, right hematometra, hematocervix, hematosalpinx, hematometrocolpos, and a strange pelvic cyst communicating with a strip-type tube located between the bladder and vagina right side. Since the resultant images were inconclusive, endoscopy was performed for evaluation. The cystoscopy revealed blood flow from the ureter opening, confirming communication between the vagina and the bladder. Simultaneously, the colpo-hysteroscopy detected two vaginal septa and cervical duplication. Finally, the abnormal anatomical structure of the urogenital system of the patient was identified using a cystoscope, ureteroscope, and laparoscope. In summary, pelvic ultrasound and MRI are valuable tools for diagnosing and categorizing female urogenital abnormalities. MRI and endoscope are crucial to diagnose complicated urogenital malformation.

### 2.4 The clinical classification of HWWS

Appropriate classifications can guide precise diagnosis and treatment. Currently, there is no unified objective and comprehensive classification system for HWWS. Four approaches have been suggested to categorize female genital tract malformation. The American Fertility Society classification of Mullerian anomalies [26], embryological-clinical classification system of genito-urinary malformations [27,28], vagina cervix uterus adnex-associated malformation (VCUAM) classification [29], and CONgenital UTerine Anomalies (CONUTA) classification system established by the ESHRE and ESGE all recognize the clinical importance of female genital anomalies (Table 2) [30], but do not specifically address HWWS.

**Table 2:** The CONUTA classification system establied by the ESHRE and the ESGE.

	classification	Sub-classification
Kidney	K_1L/R_: One kidney, absence of left/right kidney	
	K_2L/R_: Two kidneys, dysplasia of left/right kidney	
Uterine	U0: Normal uterus	
	U1: Dysmorphic uterus	U1a: T-shaped uterus
		U1b:Uterus Infantilis
		U1c: Others with minor deformities of the uterine cavity
	U2: Septate uterus	U2a: Partial septate uterus
		U2b: Complete septate uterus
	U3: Bicorporeal uterus	U3a: Partial bicorporeal uterus
		U3b: Complete bicorporeal uterus
		U3c: Bicorporeal septate uterus
	U4: Hemi-uterus	U4a: Hemi-uterus with a rudimentary (functional) cavity
		U4b: Hemi-uterus without rudimentary (functional) cavity
	U5: Aplastic uterus	U5a: Aplastic uterus with a rudimentary (functional) cavity
		U5b: Aplastic uterus without a rudimentary (functional) cavity
	U6: Unclassified malformations	
Cervix	C0: Normal cervix	
	C1: Septate cervix	
	C2: Double “normal” cervix	
	C3: Double cervix with unilateral cervical aplasia/dysplasia	
	C4: Cervical aplasia incorporates all cases of complete cervical aplasia but also those of severe cervical formation defects	
Vagina	V0: Normal vagina	
	V1: Longitudinal non-obstructing vaginal septum	
	V2: Longitudinal obstructing vaginal septum	
	V3: Transverse vaginal septum/imperforate hymen	
	V4: Vaginal aplasia	

**Fig. 4:**
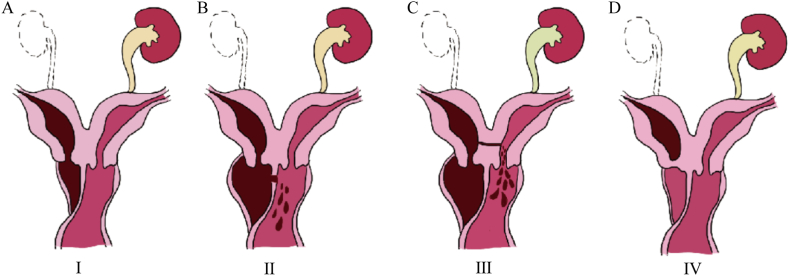
The COGA issued an expert consensus on modifying the HWWS into four classes in 2021. (A) Complete obstruction with blind hemivagina. (B) Incomplete obstruction with a communicant vaginal septum. (C) Incomplete obstruction with blind hemivagina and communicating cervix. (D) Complete obstruction with cervicovaginal atresia without communicating uteri.

In China, the Chinese Obstetricians and Gynecologists Association (COGA) issued an expert consensus on diagnosing and treating obstructive uterine and vaginal dysplasia and modified the HWWS into four classes in 2021 (Table 3, Fig. 4A–D) [31]. Nevertheless, the characteristics of our case cannot be comprehensively summarized. Another rare variant of HWWS was reported in 2022. The patient was diagnosed with HWWS alongside a complete septate uterus, left kidney ectopic dysplasia, left ectopic ureter, and three oblique vagina [32]. This research introduced a new classification method for describing the characteristics of this disorder, known as the VCUAM system [29] and the CONUTA classification system established by the ESHRE and the ESGE (KUU’V) classification [30]. This classification provides a complete summary of the pathological characteristics of HWWS, focusing on the kidney, ureter, uterus, and vaginal septum. In this system, the ectopic ureter communicates with the cervix, vagina, or perineum was presented. Herein, we introduced the KUU’CV classification to summarize the pathological features of HWWS based on the four-group classification system [33] and KUU’V classification [32]. This classification focuses on the kidney, ureter, uterus, cervix, and vaginal septum (Table 4). According to the KUU’CV classification, our case can be classified as K_2R_U_2R + V_ U′_1_ C_1_V_1_. The new classification provides a more comprehensive understanding of HWWS and can effectively reduce misdiagnosis and missed diagnosis, especially when combined with complicated urinary malformation.

**Table 3:** The classification system of HWWS in China.

Classification	Classification in 1985	Classification in 2015	Classification in 2021
Complete obstruction with blind hemivagina	Ⅰ	1.1	Ⅰ
Incomplete obstruction with communicant vaginal septum	Ⅱ	2.1	Ⅱ
Incomplete obstruction with blind hemivagina and a communicating cervix	Ⅲ	2.2	Ⅲ
Complete obstruction with cervicovaginal atresia without communicating uteri		1.2	Ⅳ

**Table 4:** KUU’CV classification system.

K(Kidney)	K_1L/R_: One kidney, absence of left/right kidney	
	K_2L/R_: Two kidneys, dysplasia of left/right kidney	
U (Ureter)	U_1L/R_: One ureter, absence of left/right ureter	
	U_2L/R_: Two ureters, ectopia of left/right ureter	U_2L/R + C_: communicates with cervix
		U_2L/R + V_: communicates with vagina
		U_2L/R + P_: communicates with perineum
U(Uterus)	U’_1_: Uterus didelphys	
	U’_2_: Septate uterus	
	U’_3_: Bicornuate uterus	
	U’_4_: Other malformations	
C(Cervix)	C_1_: Two “normal” cervix	
	C_2_: With communicating cervix C_3L/R_: Left/right cervix Atresia	
V(Vaginal septum)	V_1_: Vaginal septum without an opening	
	V_2_: Vaginal septum with an opening	

### 2.5 Treatment

This surgical management primarily aimed to alleviate symptoms, avoid complications, restore the functionality of the urogenital system, and improve fertility potential [34]. It has been reported that in a series of 51 patients with HWWS, 85,71 % were willing to achieve pregnancy and got pregnant following treatment [35]. A recent systematic review analyzed 94 studies, including 1673 patients with HWWS, and found that vaginal septum excision was recommended for a definitive diagnosis [2]. Secondary complications can result in irreversible impairment if untreated. The surgical path included transvaginal surgery, laparoscopy, or hysteroscopy. The former approach was adopted by most surgeons, and the latter approach was applied to those who had no history of sexual intercourse and desire to preserve the hymen [2,36]. Some surgeons have applied new surgical approaches, including laser and vaginal endoscopy, to treat this disorder [13]. These approaches are efficient, and most patients experience relief following these procedures. Therefore, the prerequisite is to comprehensively assess the obstruction location and anatomy of urogenital system dysplasia before surgery. The dysplastic kidney and ectopic ureter must be removed in the presence of an ectopic ureteral opening to prevent postoperative urinary incontinence [37]. In this case, before excising the vaginal septum, determining the location of the ectopic ureteral opening and its relationship with the reproductive system is important. Thus, the surgery should be performed in steps.

For complicated HWWS, gynecologists and urologists must usually perform the surgery jointly. According to our experience, complete gross and gynecological examination, preoperative comprehensive imaging examination, endoscopic evaluation (laparoscopy, colposcopy, cystoscopy, and ureteroscopy), and placement of stents along the fistula under ureteroscopy can help surgeons to locate and reduce the difficulty of surgery. A study has reported applying indocyanine green for fluorescence imaging under laparoscopy to identify hypoplasia kidney and ectopic ureters, thereby reducing the difficulty of surgery [38]. Besides this, the surgeon's experience and comprehensive understanding of embryonic development are also important for diagnosing and treating complex genitourinary malformations.

## Ethics approval and consent to participate

The Ethical Committee of the Second Hospital of Hebei Medical University approved the study, with all patients signing informed consent.

## Patient consent for publication

The patients signed an informed consent for data and image publication in this case report.

## Funding

The research received funding assistance from the 10.13039/501100001809National Natural Science Foundation of China (No. 8167060210) and the Improved Innovation Ability of the Hebei Obstetrics and Gynecology Clinical Medicine Research Center (20577705D).

## Data availability statement

All data presented in this manuscript will be made available upon reasonable request from the corresponding author.

## CRediT authorship contribution statement

**Xiaotong Xu:** Writing – original draft, Methodology, Data curation. **Yanpeng Tian:** Writing – original draft, Formal analysis. **Jingwen Zhou:** Visualization, Software, Methodology, Formal analysis. **Zhongkang Li:** Methodology, Formal analysis. **Li Meng:** Methodology, Formal analysis, Data curation. **Xianghua Huang:** Methodology, Funding acquisition, Conceptualization. **Mingle Zhang:** Methodology, Formal analysis, Conceptualization.

## Declaration of competing interest

The authors declare that they have no known competing financial interests or personal relationships that could have appeared to influence the work reported in this paper.
